# Renal Alterations Secondary to Visceral Leishmaniasis: A Scoping Review

**DOI:** 10.1590/0037-8682-0190-2025

**Published:** 2026-02-26

**Authors:** João Victor Corrêa e Silva, Lethícia de Oliveira Carvalho, Luiz Henrique da Silva, Marcos Antonio da Silva Barbosa, Pedro Vinicius Leite de Sousa, Laurisson Albuquerque da Costa

**Affiliations:** 1 Universidade Federal de Alagoas, Departamento de Medicina, Arapiraca, AL, Brasil.; 2 Universidade Federal de Jataí, Departamento de Medicina, Jataí, GO, Brasil.; 3 Universidade Federal de Sergipe, Hospital Universitário de Sergipe, Aracaju, SE, Brasil.

**Keywords:** Visceral leishmaniasis, Kidney disease, Acute kidney injury

## Abstract

**Background:**

Visceral leishmaniasis (VL) is an endemic and neglected disease in several countries. In addition to affecting the visceral tissues, kidney alterations are recurrent and result from the pathophysiological implications of infection or drug action during treatment**.** This review mapped and described renal changes, renal biomarkers, and treatment-related nephrotoxicity in humans with confirmed VL.

**Methods:**

This scoping review was conducted in accordance with the Joanna Briggs Institute Manual for Evidence Synthesis and the PRISMA-ScR guidelines. PubMed, SciELO, ScienceDirect, Scopus, and Web of Science. were searched with no temporal or geographical restrictions. We included studies involving humans with confirmed VL that reported renal alterations, kidney injury biomarkers, or treatment-related nephrotoxicity. Two reviewers independently screened the records and extracted data. Study selection was documented using the PRISMA-ScR flowchart.

**Results:**

In total, 1,444 studies were identified, of which 15 met the eligibility criteria. The included studies reported a wide spectrum of renal alterations in VL, including urinary abnormalities, kidney injury biomarkers, and acute kidney injury. Glomerular and tubulointerstitial changes, such as mesangial alterations, membranoproliferative patterns, cryoglobulinemia, interstitial nephritis, and impaired urinary concentrating ability, have also been described. Reports have also addressed the potential treatment-related nephrotoxicity.

**Conclusion:**

This scoping review identified diverse renal alterations in patients with VL, ranging from functional changes and urinary abnormalities to glomerular and tubulointerstitial involvement. Evidence has also described biomarkers of kidney injury and potential treatment-related nephrotoxicity. The heterogeneity and limited data highlight the need for robust research to clarify the underlying mechanisms, diagnostic markers, and optimal management.

## INTRODUCTION

Leishmaniasis is a disease comprising three different syndromes caused by more than 20 species of protozoa belonging to the genus *Leishmania* that can infect humans. The primary mode of transmission is the bites of infected phlebotomine sand flies^1^. Clinical manifestations vary depending on the protozoan species. For instance, *Leishmania donovani* and *L. infantum* cause visceral leishmaniasis (VL), primarily affecting the liver and spleen^2^.

Regardless of the clinical manifestations, leishmaniasis is endemic to multiple regions worldwide, with VL being the second most common form. The estimated annual incidence ranges from 50,000 to 90,000, with the highest incidences found in Brazil, East Africa, and India^3^ ^,^ ^4^. Data from Brazil’s notification system (Sistema de Informação de Agravos de Notificação) from 2018 to 2022 showed a decline in the incidence of VL; however, in 2022, there were 1,983 new cases^5^. 

Although globally relevant, VL primarily occurs in low- and middle-income countries. As with other neglected diseases, VL often receives insufficient attention, and delays in diagnosis and treatment initiation contribute to clinical complications associated with the disease^6^. Clinical manifestations include hepatosplenomegaly, anemia, pancytopenia, and hypergammaglobulinemia. However, clinical manifestations of VL can also result from damage to other organs such as the kidneys, which are commonly affected during the course of the disease^7^.

Several studies have described renal involvement in VL, including immune complex-mediated injury, hypergammaglobulinemia, cytokine-mediated damage, amyloid deposition, and drug-related nephrotoxicity. However, these manifestations have been reported across highly heterogeneous study designs, ranging from isolated case reports to small observational cohorts, which limit our understanding of the full spectrum of renal alterations^6^ ^,^ ^8^.

Although kidney damage is usually not the main cause of death in VL, individuals may develop acute kidney injury (AKI) or even chronic kidney disease, both of which significantly increase the morbidity and sequelae associated with VL^8^. However, systematized data on VL is lacking, highlighting the need for syntheses that organize and integrate the existing evidence. In this context, this scoping review aimed to map and describe renal alterations, kidney injury biomarkers, and treatment-related nephrotoxicity in humans with confirmed VL across clinical settings. This study aimed to identify gaps that may guide future research and clinical decision-making.

## METHODS

### Type of study

This scoping review followed the methodological recommendations of the Joanna Briggs Institute (JBI) Manual for Evidence Synthesis and was reported in accordance with the Preferred Reporting Items for Systematic Reviews and Meta-Analyses extension for Scoping Reviews (PRISMA-ScR) guidelines^9^.

### Review questions

A guiding question was formulated to support the development of this scoping review. This question was structured using the Population, Concept, Context (PCC) framework, as detailed below:

***Population:*** humans with laboratory-confirmed VL.

***Concept:*** renal alterations (glomerular and tubulointerstitial alterations, AKI, and electrolyte disorders), kidney injury biomarkers (e.g., neutrophil gelatinase-associated lipocalin [NGAL] and kidney injury molecule-1 [KIM-1]), and treatment-related nephrotoxicity.

***Context:*** any clinical setting across any global regions without temporal restrictions.

Therefore, the main question was, “What renal alterations, kidney injury biomarkers, and treatment-related nephrotoxicity have been reported in humans with confirmed VL in any clinical setting?” An additional sub-question was included: “What are the main clinical outcomes reported in this population?” This scoping review was registered with the Open Science Framework under the following DOI: 10.17605/OSF.IO/TGP79**.**

### Eligibility criteria

This scoping review included studies addressing patients of all age groups with VL confirmed through definitive parasitological or molecular methods (e.g., PCR or tissue aspiration examination). Clinical trials (randomized and non-randomized) and analytical or descriptive observational studies (cohorts, case series, and case reports) published up to December 7, 2025, were included without language, geographic, or temporal restrictions. 

Exclusion criteria included editorials, letters to the editor, conference abstracts, and book chapters. To ensure diagnostic reliability, studies that defined VL exclusively using clinical epidemiological criteria were excluded. Furthermore, studies addressing coinfections (e.g., HIV, hepatitis, or Chagas disease) or compromised immune status (e.g., kidney transplant recipients) were excluded to avoid confounding renal outcomes. Notably, conditions such as HIV infection can alter renal function independent of VL^10^ or exacerbate VL-associated renal alterations^11^. Finally, articles with incomplete data were excluded from the analysis.

### Search strategy

PubMed, SciELO, ScienceDirect, Scopus, and Web of Science were searched between April 1 and June 30, 2024, with updated searches in December 2024 and December 2025. Temporal or geographical restrictions were not imposed. The keywords and controlled terms were combined using Boolean operators. The following search strings were used:

“visceral leishmaniasis” AND “kidney disease”

“visceral leishmaniasis” AND “acute kidney injury

“visceral leishmaniasis” AND “glomerulonephritis”

Full search strategies for each database are provided in the Supplementary Material **.**

For the analysis, open-access articles were selected as well as those made available through the institutional access platform Federated Academic Community, accessible via the Coordination for the Improvement of Higher Education Personnel platform, which reported renal function data, including baseline status, clinical evolution, and outcomes, with an assessment of the degree of dysfunction and its specificity to the pathophysiology of the disease or nephrotoxicity. 

### Analytical Process

The retrieved references were exported to Zotero and State of the Art through Systematic Review software for automatic and manual duplication. The consolidated list was transferred to Google Sheets for blinded screening, which was conducted in two consecutive stages.

**FIGURE 1:**
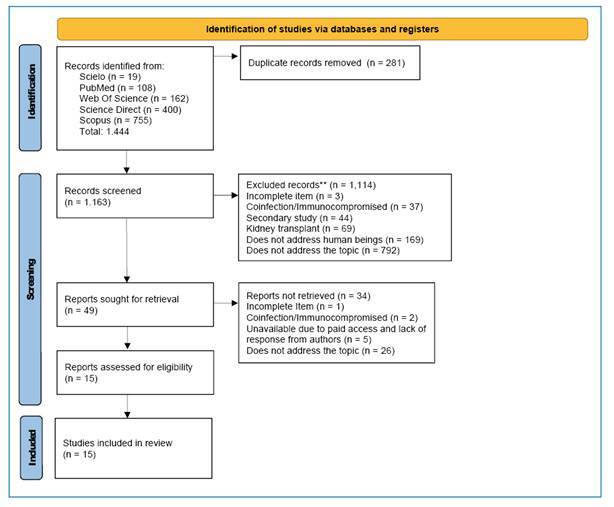
Preferred Reporting Items for Systematic Reviews and Meta-Analyses (PRISMA) 2020 flowchart showing the process of article identification, screening, eligibility assessment, and inclusion. The flowchart details the number of records identified, excluded, and included at each stage of the scoping review. Adapted from PRISMA flowchart (Page, 2021)^38^.

In the first phase (title and abstract screening), two researchers independently evaluated each reference and classified them as ‘included,’ ‘excluded,’ or ‘uncertain.’ In the second phase (full-text assessment), the pre-selected articles were reviewed in full by independent reviewers to confirm their eligibility. The selection process is documented in the PRISMA-ScR flowchart^12^ shown in Figure 1. Disagreements were resolved by discussion or consultation with a third senior reviewer when necessary.

The levels of evidence (LE) was classified for descriptive purposes only. The LE hierarchy was used as described by Wallace *et al.* (2022)^13^, as per the list below:

- Level 1: Systematic reviews or meta-analyses of randomized controlled clinical trials.
- Level 2: At least one well-designed randomized controlled trial.
- Level 3: Well-designed clinical trials without randomization.
- Level 4: Well-designed cohort and case-control studies.
- Level 5: Systematic reviews of descriptive and qualitative studies.
- Level 6: A single descriptive or qualitative study.
- Level 7: Opinions of authorities or reports of expert committees.

### Ethics

This review used only previously published research; therefore, ethical approval was not required.

### Extraction

In total, 1,444 studies were identified, 281 of which were excluded as duplicates. After title and abstract screening, 1,114 studies were excluded; among these, 792 did not address the central topic, and three had incomplete data, 37 included individuals with coinfection or immunocompromised status, 169 were not performed in humans, and 69 addressed kidney transplantation. This resulted in the assessment of 49 full-text articles for eligibility. After this stage, five studies were excluded due to lack of access, one had incomplete information, 26 did not address the research question, and two included individuals with coinfection or immunocompromised status. The final analysis included 15 studies, as detailed in the PRISMA-ScR flowchart (Figure 1).

The extraction was designed to capture information regarding study characteristics, such as authors, study design, year of publication, country of publication, objectives, sample size, and main findings. Considering renal outcomes, evidence regarding clinical and laboratory manifestations, biomarkers, tubulointerstitial and glomerular compartment modifications, AKI, treatment toxicity, and management were collected. 

## RESULTS

### Characteristics of the studies

Among the 15 included studies, publications ranged from 1972^14^ to 2025^15^, with seven published in the last decade. Nine studies were conducted in Brazil, with others from China, Iran, Spain, Somalia, and Sudan. A total of 1,209 individuals with VL were analyzed. The studies ranged from case reports to cohort studies, and described demographic data, urinary abnormalities, kidney function parameters, histological findings, AKI occurrence, treatment exposure, and outcomes (Table 1).

**TABLE 1:** Characterization of selected studies on kidney alterations associated with visceral leishmaniasis.

Authorship	Study Type	Country	Study objectives	Sample and Findings	NE
Andrade *et al*., 1972. ^14^	Case Series	Brazil	To describe the histological findings of 18 necropsy protocols whose patients were diagnosed with VL.	18 individuals/ Fibrillary thickening was observed in the mesangial region, with no involvement of the basement membrane or cell proliferation; mesangial alteration was the most prominent finding.	VI
Salgado Filho et al., 2003.^16^	Case Series	Brazil	11 patients with VL were prospectively followed to verify the clinical and laboratory repercussions of renal function in the disease.	11 individuals/ Urinalysis revealed hematuria in 63.6%, and nephritic syndrome was identified in one case, proteinuria in 90.9% patients, and leukocyturia in 54.5% of cases. Proximal tubulopathy, assessed by retinol-binding protein levels, was observed in 45.4% of cases. Microalbuminuria was observed in 81.8% of the cases.	VI
Daher *et al*., 2008.^17^	Cohort study	Brazil	To analyze the course of the disease and its clinical manifestations in 57 patients, stratified according to the serum creatinine value (<1.3 mg/dL and >1.3 mg/dL).	A total 57 individuals were analyzed, with a mean age 28 ± 18 years. Male patients predominated (74%); The average hospitalization duration was 21 ±12 days; AKI was observed in 15 patients (26.3%); all individuals in the group with sCr >1.3 mg/dL required a second medication, with eight developing AKI prior to amphotericin B administration; Greater severity was observed in the group with sCr >1.3 mg/dL. There were three deathsin the AKI group.	IV
Elnojomi *et al*., 2010.^28^	Cohort study	Sudan	To estimate the prevalence of renal injury among patients with VL and to examine its relationship with microalbuminuria.	88 individuals/All patients initially presented with serum urea and creatinine levels within normal limits. Albuminuria was detected in 35/88 (40%) of the patients with VL using ELISA test, and in 42% of cases when assessed by turbidometry.	IV
Oliveira *et al*., 2010. ^22^	Cohort study.	Brazil	To evaluate risk factors for AKI in patients with VL.	Of 264 patients diagnosed with VL, 76 (33.9%) developed AKI. The majority were men. At the admission, 26 patients presented with serum creatinine > 1.4 mg/dL, 39 developed AKI after starting treatment with amphotericin B, seven after initiation of glucantime treatment, and four due to comorbidities.	IV
Daher *et al*., 2013.^18^	Cohort study	Brazil	To describe the clinical manifestations, laboratory tests, and evolution of patients with VL and AKI admitted to a reference ICU in Northeastern of Brazil.	Among 253 patients admitted to the ICU over 6 years, 10 patients (4%) developed AKI. All were men; length of hospitalization ranged from 7 to 303 days (61.2 ± 87.9 days), with mean serum urea 105.5 ±27.6 mg/dL and serum creatinine 2.7±1.1 mg/dL. Oliguria was observed in six cases. According to the RIFLE criteria AKI was observed in two patients without coinfection.	IV
Rocha *et al*., 2013.^26^	Cohort study	Brazil	To compare clinical manifestations, morbidity and mortality between adults and children with VL and focus in kidney function	Among 432 patients with VL, divided into individuals aged > 21 years and ≤ 21 years, AKI was observed in 160 (37%) individuals, in a similar proportion between the two groups. Eleven adults required hemodialysis. In the pediatric population, secondary infection was a risk factor for AKI, whereas in the adult population, hypokalemia, leukopenia, chills, and the use of amphotericin B were associated with AKI. Mortality was not related to AKI.	IV
Oliveira *et al*., 2014.^25^	Cohort study	Brazil	To investigate the association between kidney irregularities and biomarkers of inflammation in VL.	Sixteen patients with VL before treatment were compared with 13 healthy individuals and five patients with VL after treatment. All the VL cases presented concentration deficit before treatment, and 80% after treatment. Urinary acidification deficit was observed in 56.2% of the cases before treatment and 40% after treatment. Urinary inflammation biomarkers monocyte chemotactic protein-1 and malondialdehyde were higher in the VL group.	IV
Ortiz, M. *et al*., 2015.^23^	Case series	Spain	To describe a series of cases attempting to associate glomerulonephritis and cryoglobulinemia as the first manifestation of VL.	Two cases without coinfection. Case report 2 presented hematuria, proteinuria 20 g/day, and rapidly progressive renal dysfunction. Renal histology revealed cryoglobulinemic MPGN with subendothelial deposits and epithelial crescents in more than 80% of glomeruli. The patient's renal condition deteriorated after treatment with immunosuppressive therapy, but subsequently improved after amphotericin B therapy. Case 3 presented AKI, nephrotic proteinuria, and hematuria. Histopathological findings included mesangial proliferation and increase of matrix, peripheral extension to some capillaries with double contour images. Electron microscopy confirmed MPGN, and intense tubulo-interstitial injury. Hemodialysis and liposomal amphotericin B were initiated. After treatment, renal function did not improve.	VI
Meneses et *al*.,2018.^24^	Cohort study.	Brazil	To evaluate the usefulness of early AKI biomarkers in clinical management of VL	In a cohort of 50 hospitalized patients with VL, common abnormalities included hyponatremia, hypoalbuminemia, hypergammaglobulinemia. AKI was observed in 46%, and one patient (2%) died. Serum neutrophil gelatinase-associated lipocalin sNGAL demonstrated an early association with developed of AKI. Patients in AKI group experienced significantly longer hospital stay.	IV
Romero *et al*., 2020.^19^	Case Report	Spain	To report a case of a patient with kidney injury secondary to VL.	A patient presented with sCr 2,51 mg/dL, proteinuria 1.7 g/24 hours, hematuria, and polyclonal hypergammaglobulinemia. Histology showed mesangial and endocapillary proliferation, and the capillary borders appeared with double-contour images. Crescents were evidenced in three glomeruli. The interstitium revealed the foci of inflammatory infiltrates consisting of lymphomonocyte cells. The findings were consistent with type I membranoproliferative glomerulonephritis. Mixed cryoglobulin type II was positive. Corticoids therapy was initiated; however, kidney function improved only after liposomal amphotericin B treatment initiation.	VI
Zou *et al*., 2021.^20^	Case Report	China	A case report describing persistent hypergammaglobulinemia and increased urinary protein excretion despite recovery from VL.	A patient presented with an SCr of 72.5 μmol/L and proteinuria. Sodium antimony gluconate was initiated, but the proteinuria persisted. A renal biopsy was performed, and histology showed glomerular hypertrophy with segmental mesangial alterations without tubulointerstitial involvement. Electronic microscopy revealed enlarged lysosomes in the adjacent tubules. Following treatment with valsartan, both urinary protein and serum IgG levels gradually decreased, and serum creatinine levels remained stable during the three-month follow-up.	VI
Mohamed et al., 2022.^21^	Case report	Somalia	To report a case of a patient with a significant diagnostic probability of VL with renal involvement.	A 19-year male patient presented with markedly elevated urea (228 mg/dL), creatinine levels (4.47 mg/dL) and potassium (6.58 mEq/L). Initial management included hemodialysis, and therapy with sodium antimoniate. However, the patient developed acute pancreatitis following sodium antimoniate. Treatment was switched to liposomal amphotericin B, leading to progressive normalization of laboratory parameters after initiation.	VI
Corrêa-Castro *et al*., 2024.^27^	Cohort study	Brazil	To analyze the correlation between circulating immune complex levels during VL and biological markers of AKI.	14 patients with VL were evaluated between early therapy and 12 months post-treatment (mpt). High levels of circulating immune complexes were observed in VL patients up to 6 mpt. AKI was identified in 12 patients. Plasmatic cystatin C was positively correlated with circulating immune complexes and immunoglobulins. C5a levels was associated with cystatin C, circulating immune complexes and immunoglobulins. No association between amphotericin B use and kidney function markers.	IV
Mansoursamaei *et al*., 2025.^15^	Case Report	Iran	Case report of an uncommon manifestation of VL in a non-endemic region	A 30-year male patient presented with nephrotic syndrome and AKI. Kidney biopsy revealed MPGN. He required hemodialysis and was treated with prednisone. Following the VL diagnosis, liposomal amphotericin B was initiated. Despite treatment, he remained dialysis- dependent. Five months later, he was readmitted with cardiac tamponade, which did not improve despite proper management, resulting in death.	VI

**NE:** level of evidence; **VL:** visceral leishmaniasis; **AKI:** acute kidney injury; **sCr:** serum creatinine; **MPGN:** membranoproliferative glomerulonephritis. **IV:** well-designed cohort and case-control studies; **VI:** a single descriptive or qualitative study; **RIFLE:** risk, injury, failure, loss and end-stage renal disease; **ELISA:** enzyme-linked immunosorbent assay .

### Clinical manifestations (signs and symptoms)

The main signs and symptoms included fever (the most common, with temperatures reaching up to 40 °C), anemia, hepatosplenomegaly, adenomegaly, jaundice, asthenia, anorexia, and vomiting. Additional symptoms included pancytopenia, hematuria, purpuric lesions, abdominal pain, diarrhea, and dyspnea^16^ ^-^ ^20^.

### VL diagnosis

Bone marrow aspiration was the primary method for diagnosing VL^14^ ^-^ ^27^. One study employed *Leishmania* DNA detection in urine^28^. Additionally, Enzyme-Linked Immunosorbent Assay (ELISA) was used. Although this method has limitations, the association between clinical symptoms and response to treatment with liposomal amphotericin B supported the diagnosis^21^ ^,^ ^22^.

### Main Findings

Kidney manifestations associated with VL ranged from asymptomatic urinary abnormalities to significant glomerular and tubulointerstitial changes with varying degrees of glomerular filtration rate (GFR) impairment, including severe cases that required dialysis or resulted in death^15^ ^,^ ^17^ ^,^ ^22^ ^-^ ^24^. The studies were heterogeneous and provided data on clinical manifestations, diagnostic investigations, kidney injuries, therapeutic approaches, and outcomes associated with VL (Table 2 and Table 3). The results are presented according to the renal alterations associated with VL (laboratory changes, affected renal compartments, and AKI), followed by treatment-related nephrotoxicity.

**TABLE 2:** Presentation of indicators of kidney injury and pharmacological treatments reported in the selected studies.

Study	AKI			Glomerular Alteration		Drug Therapy			
	Creatinine	Urinary output	Diagnostic criteria	Albuminuria	Kidney Biopsy	Amphotericin B	Another Drug?	Was there any post-treatment kidney evolution?	Dosage
Andrade *et al*.^14^	U	U	NAC	U	Y	U	U	U	N
Salgado et al.^16^	Y	U	Creatinine (NAC)	Y	N	U	U	U	N
Daher *et al*.^17^	Y	U	Creatinine cutoff ≥ 1.3 mg/dL (NAC)	U	U	Y	Y	Y	Antimonial: 20 mg/kg/day; 20-40 days. Amphotericin B: 7-20 mg/kg (total cumulative dose); up to 20 days (severe disease)
Elnojomi *et al*.^28^	Y	U	Microalbuminuira (NAC)	Y	N	U	U	U	N
Oliveira *et al.* ^22^	Y	Y	RIFLE	U	U	Y	Y	Y	Pentavalent antimonials (20 mg/kg/day, 20-40 days); Amphotericin B deoxycholate (7-20 mg/kg total, ≤20 days, severe cases);
Daher *et al*.^18^	Y	Y	RIFLE	N	N	U	U	U	N
Oliveira *et al.* ^25^	Y	U	NAC	Y	N	Y	N	N	Pentavalent antimonial (meglumine antimoniate, 20 mg/kg/day for 20 days);
Ortiz et al.^23^	Y	U	NAC	Y	Y	Y	N	N	U
Meneses et al.^24^	Y	N	KDIGO	Y	N	Y	Y	N	U
Romero *et al*.^19^	Y	U	NAC	Y	Y	Y	N	Y	Liposomal amphotericin B (4 mg/kg/week for 5 weeks).
Zou *et al*.^20^	Y	U	Proteinuria (NAC)	Y	Y	N	Y	Y	N
Mohamed et al.^21^	Y	U	KDIGO	U	N	Y	Y	Y	Sodium stibogluconate (800 mg/day; discontinued due to pancreatitis); Liposomal Amphotericin B (3 mg/kg IV, days 1-5, 14, 21)
Corrêa-Castro *et al*.^27^	Y	U	KDIGO	N	N	Y	Y	Y	Amphotericin B (desoxycholate, liposomal or lipid complex); regimen switches to liposomal formulation due to adverse events; daily dose 1.4-5.0 mg/kg (median 2.5 mg/kg); complete clinical remission in all patients
Mansoursamaei et al.^15^	Y	Y	NAC	U	N	Y	N	U	Liposomal amphotericin B 3 mg/kg on Days 1 to 5, 14, and 21
Rocha et al.^26^	U	Y	RIFLE	U	U	Y	Y	Y	Pentavalent antimonial (meglumine antimoniate) 20 mg/kg/day IV for 20-40 days; amphotericin B (0.7-1.0 mg/kg/day IV for 14-21 days) used in infants <6 months, adults >60 years, unstable patients, or after antimonial failure;

**U:** unspecified; **Y:** Yes; **N:** No; **AKI:** acute kidney injury; **NAC:** no AKI criteria; **RIFLE:** risk, injury, failure, loss and end-stage renal disease, **KIDGO:** Kidney Disease: Improving Global Outcomes.

**TABLE 3:** Clinical features and laboratory results of patients with visceral leishmaniasis.

	**Andrade *et al*.** ^14^	Salgado et al.^16^	**Daher *et al*.** ^17^	**Elnojomi *et al*.** ^28^	**Oliveira *et al*.** ^22^	**Daher *et al*.** ^18^	**Oliveira *et al*.** ^25^	Ortiz et al. ^23^	**Meneses et *al*.** ^24^	**Romero *et al*.** ^19^	**Zou *et al*.** ^20^	Mohamed et al.^21^	**Corrêa-Castro *et al*.** ^27^	**Mansoursamaei *et al*.** ^15^	**Rocha *et al*.** ^26^
Participants	18	11	57	88	224	4 no coinfection	29	2 no coinfection	50	1	1	1	14	1	432
Control Group	No	No	SCr<1.3 mg/dL />1.3 mg/dL	No	No AKI /AKI	No	VL group/ Healthy group	No	No AKI/ AKI	No	No	No	No	No	No
Sex		M: 8 | F: 3	M: 28	-	M:107	M	M: 15	M: 2	M: 25 F:2/ M: 18 F:5	M	M	M	M: 11 F:3	M	> 21 (n = 239)
			F: 14 / M: 14		F: 41/M: 65		F:1 /								M: 43.5%
			F:1		F: 11		M: 10 F3								F: 56.5%
															≤ 21 years (n = 193)
															M: 25.9%
															F: 74.1%
Age(years)		1-25*	18-24 / 14-37*	-	33±14/ 41±15 **	17-51	42 ± 17 / 40 ± 15 **	47; 76	39±15/ 48 ± 22 **	69	30	19	41.5 (31-46.5) ***	30	> 21: 40.8 ± 13.8 years
															≤ 8.4 ± 6.4 years
AKI	-	-	-	-	RIFLE	RIFLE-R	-	-	KDIGO	-	-	KDIGO	KDIGO	-	RIFLE-R
Diagnostic Criteria					(U)	0 (0.0%)			Stage 1: 40% (20/50)			Stage 1: 0.0%	Stage 1: 50.00% (7/14)		15 (17.9%)
						RIFLE-I			KDIGO			KDIGO	KDIGO		RIFLE-I
						2 (22.2%)			Stage 2: 6% (3/50)			Stage 2: 0.0%	Stage 2: 21.42% (3/14)		38 (45.2%)
						RIFLE-F			KDIGO			KDIGO	KDIGO		RIFLE-F
						7 (77.8%)			Stage 3: 0%			Stage 3: 100% (1/1)	Stage 3: 14.28% (2/14)		31 (36.9%)
															pRIFLE-R
															45 (60.5%)
															pRIFLE-I
															21 (31.6%)
															pRIFLE-
															F 1 (7.9%)
Albuminuria	-	30-127 mcg/min	-	Detectable	-	-	17.3 ± 23.8 / 6.7 ± 6.3 mg/day	20 g / 24h; (-)	12.8±10.4/18.2±11.8	1.7 g/ 24h	6.88 g/24 h	-	-	-	-
Hematuria	-	2-30	-	-	4.1%/5.3%	-	-	Yes; Yes	-	Yes	-	-	-	-	-
Oliguria	-	-	-	-	0%/ 16.6%	3	-	-	-	-	-	-	-		
Creatinine (mg/dL)		0.5-1.6	Maximum: 0.7 ± 0.2 / 3.6 ± 5.2	normal	0.8 ± 0.2 /1.8 ± 1.8	1.8 ± 5.0	1.0 ± 0.2 / 1.0 ± 0.2	1.6 - 6; (-)	0.89±0.2 / 1.11±0.36	2.51	1.05	4.47 - 0.56	Increased during treatment	2,5 - 4,1	-
Gamma Globulin Alteration	-	yes	-	-	-	-	-	-	Yes	Yes	Yes	-	Yes	-	-
Need for Dialysis	-	No	No	-	-	Yes - 3 cases	No	Yes; Yes	No	No	No	Yes	No	No	Yes - 11 cases
Deaths	NA	0	0 / 3	-	3.1% / 30.2%	2	0	Yes; No	0/ 1 (2%)	0	0	0	No		34

**SCr:** serum creatinine; **VL:** visceral leishmaniasis; **KIDGO:** Kidney Disease: Improving Global Outcomes; **AKI:** acute kidney injury; **M:** male; **F:** female; **NA:** not available; **U:** unspecified; **RIFLE-I:** Injury; **RIFLE-F:** Failure; **RIFLE-R:** Risk; **pRIFLE:** Pediatric RIFLE; **(-):** Not reported or not specified; **g/24 h:** grams per 24 hours; **mg/day:** milligrams per day; **mcg/min:** micrograms per minute. * Minimum and maximum; ** Mean ± standard deviation; *** Median (interquartile range). Values separated by **/** represent comparative groups within the same study.

### Renal Alteration

Regarding the key findings, VL can affect both glomerular and tubulointerstitial compartments^15^ ^-^ ^20^ ^,^ ^22^, demonstrating structural damage. AKI was reported in several studies, which also described elevated serum creatinine (sCr) levels and reduced urine output^17^ ^,^ ^18^ ^,^ ^21^ ^-^ ^23^. 

Among urinary modifications, proteinuria was a frequent finding in VL^16^ ^,^ ^19^ ^,^ ^20^ ^,^ ^28^. Initial necropsy data revealed that over 80% of individuals (n = 15, 83.3%) had morphological evidence of proteinuria^14^. Furthermore, a study performed in Sudan indicated that approximately 40% of individuals with VL had mild kidney damage, with microalbuminuria detected using ELISA (n = 35, 39.7%) and immunoturbidimetry (n = 37, 42%)^28^. 

Indeed, proteinuria is common in VL and can be associated with alterations in urinary sediments. One study reported the presence of proteinuria (n = 10, 90.9%), leukocyturia (n = 6, 54.5%), and hematuria (n = 7, 63.6%). Hypoalbuminemia and hypergammaglobulinemia were observed in all the individuals^16^. Furthermore, higher levels of proteinuria have been observed in some glomerular diseases related to VL^23^. In a case of membranoproliferative glomerulonephritis (MPGN) with cryoglobulinemia, the patient presented with proteinuria (1.7 g/24 hours), hematuria, and sCr elevated (2.51 mg/dL)^19^. 

Concerning glomerular manifestations, the most common finding was MPGN in some cases associated with cryoglobulinemia^15^ ^,^ ^19^ ^,^ ^23^. This manifestation may be due to hypergammaglobulinemia, ruling out other possible causes of renal alterations such as autoimmune diseases^19^ ^,^ ^23^. Some cases were associated with severe complications such as the need for hemodialysis or death^15^ ^,^ ^22^. Mesangial involvement was reported in some articles^14^ ^,^ ^20^.

Tubulointerstitial manifestations were also observed in patients with VL. One study demonstrated that patients with AKI and VL exhibit increased levels of urinary kidney injury molecule-1 and serum NGAL (sNGAL)^24^. Furthermore, one report showed tubulointerstitial nephritis on anatomopathological analysis^19^. One study observed proximal tubule damage, with 45.4% of the patients showing increased excretion of retinol-binding protein^16^.

Another study reported that all patients with VL exhibited urinary concentration deficits before treatment initiation. Following treatment reassessment, most patients showed persistently reduced urinary osmolality (below 700 mOsm/L), even after water deprivation and desmopressin acetate administration. Before treatment, urinary acidification was assessed, which revealed that more than half of the patients showed acidification deficits after calcium chloride (CaCl₂) overload. Additionally, urinary oxidative stress biomarker levels were higher in the VL group before treatment than in the control group^25^.

Among the studies that described AKI, the incidence ranged from 26.3% to 46%^18^ ^,^ ^21^ ^,^ ^22^ ^,^ ^24^ ^,^ ^26^ ^,^ ^27^. A study conducted in Brazil demonstrated that sNGAL could serve as a promising early biomarker of AKI^24^. Furthermore, AKI was associated with disease severity, longer hospitalization time, dialysis requirement and mortality^17^ ^,^ ^18^ ^,^ ^21^ ^,^ ^24^. Another Brazilian study showed that individuals with higher sCr levels (classified as those with sCr > 1.3 mg/dL) required a second-line drug for treatment and had a higher mortality rate^14^. 

Similarly, a cohort study of individuals with VL analyzed those with and without AKI and demonstrated that older age, male sex, and jaundice were associated with AKI (n = 76, 33.9%). Additionally, only 6.5% of the patients exhibited oliguria. Mortality was significantly higher in patients with AKI (n = 23, 30.2%) than in those without AKI (3.1%; *p* < 0.0001)^20^. Another study compared adults aged > 21 years with children aged ≤ 21 years and found a similar incidence of AKI (35.1% *vs* 39.4%, respectively)^27^. In the adults with hypokalemia, the use of amphotericin B, chills, and leukopenia were associated with AKI^15^. 

In addition, a case series (n = 4) found that among patients with VL and AKI, the median urea level was 105.5 mg/dL (86-136 mg/dL), the median sCr was 2.675 mg/dL (1.8-5.0 mg/dL), all patients were men, the median age was 36.5 years (17-51 years), and the median hospitalization time was 36 days (10-48 days)^18^.

### Treatment

Among the selected studies (n = 15), 60% (n = 9/15) reported that patients were treated with amphotericin B as a part of VL management. Of these, 26,7% of all included studies (n = 4/15) explicitly specified the liposomal formulation as amphotericin B^15^ ^,^ ^19^ ^,^ ^21^ ^,^ ^27^. When the treatment details were reported, the regimens included pentavalent antimonials and amphotericin B deoxycholate, with substantial variability in dosing schemes and treatment durations across studies (**Table 2**).

### AKI after treatment

Regarding AKI following treatment, a few studies evaluated kidney function before and after amphotericin B. One study reported that patients with VL were initially treated with intravenous pentavalent antimonials (20 mg/kg/day for 20-40 consecutive days). In severe cases, amphotericin B deoxycholate was administered at a total cumulative dose of 7-20 mg/kg over 20 days. In this cohort, 39 (51.3%) and seven (9.2%) patients developed a new episode of AKI within a mean of 10 days after initiating amphotericin B treatment and pentavalent antimonial therapy, respectively. In addition, among patients with severe VL who were unresponsive to sodium antimony gluconate, treatment with amphotericin B was significantly associated with AKI (p < 0.0001). Notably, lipid-based amphotericin B formulations were not used^22^. 

In another study, sodium antimony gluconate was used as the primary antileishmanial agent. Patients with sCr < 1.3 mg/dL were exclusively treated with pentavalent antimonial therapy. In contrast, patients with sCr > 1.3 mg/dL required adjunctive therapy, primarily amphotericin B; in a total of 15 individuals with AKI, eight developed AKI before amphotericin B treatment. All three recorded deaths occurred in the group with elevated sCr^17^.

Despite the nephrotoxicity associated with VL treatment, disease improvement is a key factor in reducing inflammation and facilitating AKI recovery^20^. One study reported a decrease in albuminuria after treatment^26^. A few case reports showed improvement following liposomal amphotericin B treatment^19^ ^-^ ^21^. Another study demonstrated a peak in sCr levels during treatment with improvements observed in the post-treatment period. However, no changes in sCr levels were recorded during amphotericin B therapy, suggesting that other factors may have contributed to the worsening of kidney function^28^. 

Regarding glomerulonephritis treatment, two cases of MPGN associated with cryoglobulinemia were treated with liposomal amphotericin B. One of these cases also received immunosuppressive therapy; however, the outcome was fatal, while the other case showed improvement in the clinical manifestations of VL but without recovery of kidney function^23^. Similarly, in a case of MPGN, the patient was treated with prednisone and hemodialysis and subsequently received liposomal amphotericin B after VL diagnosis. Despite treatment, the patient remained on HD and died five months after initial presentation^15^. In contrast, another case of MPGN associated with cryoglobulinemia showed an improvement in kidney function after treatment with liposomal amphotericin B^19^. 

In another case in which only mesangial alterations were observed without immune complex deposits, treatment with sodium antimony gluconate improved VL; however, proteinuria remained high. The addition of valsartan reduced the proteinuria^20^. Therefore, the treatment of VL is a key component of management, whereas the role of immunosuppressive therapy requires further investigation to define its benefits and risks. 

## DISCUSSION

The present study offers a summarized review of the associations between VL and kidney alterations, emphasizing the complexity of the disease and its potential impact on various kidney compartments, including the glomeruli and tubulointerstitial regions. This review highlights the high incidence of AKI, including severe cases that may necessitate dialysis or lead to death. The treatment of VL-related kidney complications is challenging owing to its association with impaired kidney function; however, intervention is crucial for alleviating the manifestations of VL and preventing further deterioration of the GFR.

First, the incidence of AKI was notable, although it was comparable to that observed in other neglected infectious diseases^29^. Despite the high incidence and severity of AKI, data on AKI in VL remain limited. Research on early diagnosis and clinical management of AKI is scarce, highlighting the urgent need to allocate resources to address this important complication of VL. Notably, some studies were conducted in hospital settings, and, in some cases, in intensive care units, where individuals tend to present with more severe cases^18^. 

Although the criterion proposed by Kidney Disease: Improving Global Outcomes (KDIGO) is currently the most used and widely accepted in clinical practice, as well as in research, its application only became widespread over the last decade^30^. Many of the included studies were conducted before KDIGO implementation, limiting data standardization. Nevertheless, these studies have provided clinically relevant insights. For instance, NGAL has shown potential as an early AKI biomarker^25^. Several studies have identified this biomarker as an effective indicator of kidney damage^31^ ^,^ ^32^. Furthermore, consistent with numerous previous studies, AKI was associated with disease severity, as reflected by longer hospital stays, need for additional medications, and increased mortality^33^ ^,^ ^34^.

Regarding glomerular manifestations, all the included studies were case reports or case series, and both MPGN and mesangial alterations were observed. Other studies on parasitic diseases have reported comparable findings, which may be pathophysiologically related to immune complex deposition^35^. Some case reports have described other glomerular manifestations such as amyloidosis^36^. However, such data may be absent from this review owing to the decision to exclude articles describing coinfections and immunosuppressive conditions to focus specifically on the role of VL in kidney manifestations. Presentations such as amyloidosis and other glomerular diseases may occur more frequently in these excluded conditions. Therefore, further studies are required to explore this issue.

Treatment of VL is primarily based on amphotericin B, although some studies have also considered pentavalent antimonials. The toxicity of these medications differs in the context of a reduced GFR. Pentavalent antimonials are predominantly eliminated by the kidneys; consequently, toxicity, particularly cardiotoxicity and pancreatitis, increases in individuals with reduced GFR^37^. In contrast, amphotericin B is potentially nephrotoxic, mainly due to tubular damage and reduced renal blood flow^38^. As most studies were conducted in Brazil, this finding is consistent with the guidelines for the treatment of VL in the Americas, which strongly recommend the use of liposomal amphotericin B. In contrast, pentavalent antimonials or deoxycholate amphotericin B have conditional recommendations^39^. The management of glomerulonephritis associated with VL remains uncertain and may involve either the exclusive treatment of VL or a combination of immunosuppressive therapy and supportive treatment with antiproteinuric agents. However, further studies are required to address this issue.

This study had several limitation. First, most studies were observational or case reports. Finally, despite the limited data, this review synthesized renal manifestations and treatment. Therefore, this underscores the need to focus efforts on increasing and improving research in this area and achieving the identification of evidence regarding the potential of VL to directly cause kidney manifestations by itself.

## CONCLUSION

Based on the available evidence, VL has been reported to be potentially associated with renal alterations, including histological kidney changes, laboratory abnormalities, and AKI, either associated with infection or secondary to treatment. This review mapped the reported risk groups, renal clinical manifestations, and informed early management strategies, underscoring the importance of monitoring renal alterations and searching for validated biomarkers that improve early detection in this population. 

However, several gaps were identified that may help guide future research in the field of AKI. Because both VL and its treatment are potential contributors to AKI, prospective studies with better differentiation between AKI occurring before treatment, after treatment initiation, and at long-term follow-up are essential. In the field of glomerular involvement, additional studies including a larger number of individuals with glomerulonephritis are needed to better characterize the main histological alterations and define management strategies, including VL treatment alone or in combination with immunosuppressive therapy. Such efforts would strengthen the evidence base for optimal management strategies by identifying serum and renal parameters associated with worse outcomes and potentially reduce morbidity and mortality.

## Data Availability

The research data are available in the Open Science Framework (OSF) repository at the following link: https://archive.org/details/osf-registrations-tgp79-v1
