# Supplementary material for: Renal Alterations Secondary to Visceral Leishmaniasis: A Scoping Review
Source: Rev Soc Bras Med Trop. 2026 Feb 26;59:e0190-2025. doi: 10.1590/0037-8682-0190-2025 (PMC13037720; doi:10.1590/0037-8682-0190-2025)
Supplement: Supplementary material [file 1678-9849-rsbmt-59-e0190-2025-md1.pdf]

SUPPLEMENTARY MATERIAL – SEARCH STRATEGY: TRANSPARENCY AND REPRODUCIBILITY

Review title: Renal Alterations Secondary to Visceral Leishmaniasis: An Integrative Review

1. SCIELO:

Initial search period:	April 1, 2024 to June 30, 2024.
Update:	December 2024.
Final review data:	December 2025.
Restrictions:	No geographical, language or data limits were used in the search
Boolean logic and MeSH/DeCS:	The Health Sciences Descriptors and Boolean Operator applied in the database were “Visceral Leishmaniasis AND Kidney Disease,” “Visceral Leishmaniasis AND Acute Kidney Disease,” and “Visceral Leishmaniasis AND Glomerulonephritis.”

2. PUBMED:

Initial search period:	April 1, 2024 to June 30, 2024.
Update:	December 2024.
Final review data:	December 2025.
Restrictions:	No geographical, language or data limits were used in the search
Boolean logic and MeSH/DeCS:	The Health Sciences Descriptors and Boolean Operator applied in the database were “Visceral Leishmaniasis AND Kidney Disease,” “Visceral Leishmaniasis AND Acute Kidney Disease,” and “Visceral Leishmaniasis AND Glomerulonephritis.”

3. SCIENCE DIRECT:

Initial search period:	April 1, 2024 to June 30, 2024.
Update:	December 2024.
Final review data:	December 2024.
Restrictions:	No geographical, language or data limits were used in the search
Boolean logic and MeSH/DeCS:	The Health Sciences Descriptors and Boolean Operator applied in the database were “Visceral Leishmaniasis AND Kidney Disease,” “Visceral Leishmaniasis AND Acute Kidney Disease,” and “Visceral Leishmaniasis AND Glomerulonephritis.”

4. WEB OF SCIENCE:

Initial search period:	April 1, 2024 to June 30, 2024.
Update:	December 2024.
Final review data:	December 2025.
Restrictions:	No geographical, language or data limits were used in the search

Boolean logic and MeSH/DeCS:	The Health Sciences Descriptors and Boolean Operator applied in the database were “Visceral Leishmaniasis AND Kidney Disease,” “Visceral Leishmaniasis AND Acute Kidney Disease,” and “Visceral Leishmaniasis AND Glomerulonephritis.”

5. SCOPUS:

Initial search period:	April 1, 2024 to June 30, 2024.
Update:	December 2024.
Final review data:	December 2025.
Restrictions:	No geographical, language or data limits were used in the search
Boolean logic and MeSH/DeCS:	The Health Sciences Descriptors and Boolean Operator applied in the database were “Visceral Leishmaniasis AND Kidney Disease,” “Visceral Leishmaniasis AND Acute Kidney Disease,” and “Visceral Leishmaniasis AND Glomerulonephritis.”
